# 

**Published:** 1855-01

**Authors:** 


VOL. II.] DEC. & JAN., 1854-5. [NO. II.

### IOWA

### MEDICAL JOURNAL.

CONDUCTED BY THE FACULTY OF THE

MEDICAL DEPARTMENT OF THE IOWA UNIVERSITY.

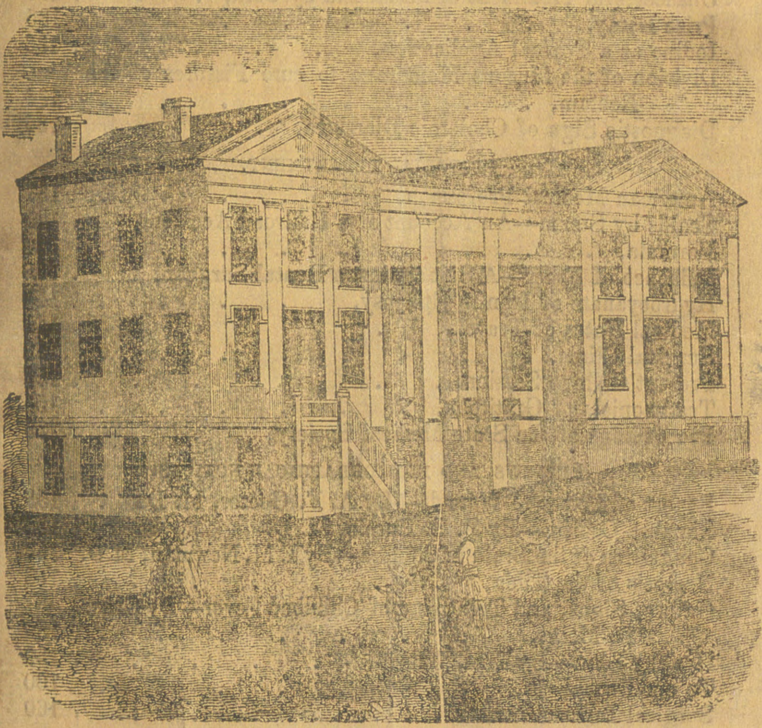

TERMS—TWO DOLLARS, IN ADVANCE.

KEOKUK, IOWA:

PRINTED AT THE WHIG BOOK AND JOB OFFICE.

Address all Communications, post-paid, to "Medical College, Box 109, Keokuk, Iowa.

